# Use of Ghrelin as a Treatment for Inflammatory Bowel Disease: Mechanistic Considerations

**DOI:** 10.1155/2011/189242

**Published:** 2011-08-09

**Authors:** Mark D. DeBoer

**Affiliations:** Department of Pediatrics, Division of Pediatric Endocrinology, University of Virginia School of Medicine, P.O. Box 800386, Charlottesville, VA 22908, USA

## Abstract

Inflammatory bowel diseases (IBD)—and in particular Crohn's disease—are immune-mediated processes that result in denuded intestinal mucosa and can produce decreased appetite, weight loss, and systemic inflammation. Current treatments include anti-inflammatory medications, immunomodulators, and feeding interventions. Ghrelin is an endogenous orexigenic hormone that directly stimulates growth hormone release, increases gut motility, and has cardiovascular and anti-inflammatory properties. Although ghrelin levels are elevated in active IBD, administration of ghrelin in most (but not all) animal models of colitis has produced improvements in disease activity and systemic inflammation. The mechanism for these effects is not known but may relate to decreased inflammation, increased motility, increased appetite, and increased colonic blood flow. Human trials have not been performed, however, and more research is clearly needed.

## 1. Introduction

Inflammatory bowel diseases (IBD)—including Crohn's disease and ulcerative colitis—are immune-mediated processes that result in denuding of intestinal mucosa [1]. Treatment largely focuses on targeting local and systemic inflammation, including via administration of steroids, immunomodulating agents, and biologic medications such as monoclonal antibodies to inflammatory cytokines [2]. Alternate treating regimens include nutritional interventions in which high-calorie feedings are given via nasogastric tube, which—despite less frequently used—have been also shown to be effective [3].

One intriguing agent that has been shown to ameliorate IBD in animal models is the orexigenic hormone ghrelin [4–6]. Following initial identification in 1999, ghrelin has attracted extensive attention for its role in physiology and its potential role as a treatment in disease states [7]. Ghrelin is released predominantly by the endocrine cells of the stomach and acts on appetite-regulating centers in the hypothalamus to stimulate increased food intake [8]. Ghrelin also acts at the level of the pituitary to stimulate growth hormone release [4]. Interestingly, ghrelin also has been shown to have anti-inflammatory [9–11] and prokinetic properties [12, 13]. It remains unclear which of these properties is responsible for its efficacy in animal models of IBD.

This paper will focus on data regarding the physiology of ghrelin in the setting of IBD, its effects in the setting of animal models of IBD treatment, and potential mechanisms whereby ghrelin may act to improve pathophysiology of IBD. As we will see, though the exact mechanism of ghrelin's action in IBD is uncertain, its efficacy in reducing local inflammation in animal models warrants consideration as a possible future treatment of IBD in humans.

## 2. Ghrelin Physiology

While small amounts of ghrelin are expressed in the hypothalamus, small intestine, and other tissues, ghrelin is produced primarily by gastric endocrine cells. Ghrelin is traditionally thought of as a meal-initiating hormone, given that levels increase just prior to meals and fall after meals [14], though recent research has implicated the presence of C6–C10 fatty acids in the diet as a positive regulator of ghrelin secretion [15]. Ghrelin secretion also may be influenced by levels of leptin, a marker of long-term energy reserves secreted by adipocytes, in that obese individuals exhibit an inverse correlation between ghrelin and leptin [16]. Finally, the role of inflammation in the regulation of ghrelin release remains unclear: ghrelin levels are increased among septic patients in the intensive care unit compared to controls [17]; however, administration of lipopolysaccharide to rodents, triggering an increase in systemic inflammation, results in a decrease in ghrelin secretion [18, 19]. 

Ghrelin is the only O-octanoylated peptide in the body, with a preference for C8 fatty acids added to a serine moiety by the enzyme ghrelin O-acyl transferase (GOAT) [20, 21]. The acylated (octanoylated) form of ghrelin has a serum half-life of only 30 minutes because of rapid metabolism to a des-acylated form that is more stable in serum [22]. Acyl-ghrelin binds to the growth hormone secretagogue-receptor 1a (GHSR-1a) [23] in widespread tissues to produce multiple effects [24].

1. *Growth Hormone Stimulation*. Ghrelin acts on receptors in the pituitary to directly stimulate growth hormone release [4].
2. *Appetite Stimulation and Adipogenesis*. Ghrelin acts on energy-regulating centers in the hypothalamus to increase appetite [8, 25] and on adipose tissue to promote adipogenesis [26].
3. *Cardiovascular Effects*. Ghrelin acts on cardiac and endothelial tissue to increase cardiac output and decrease blood pressure [27–29].
4. *Anti-Inflammatory Effects*. Ghrelin acts on receptors on lymphocytes to produce anti-inflammatory effects, including reduction of circulating cytokines [9–11, 30, 31].
5. *Intestinal Motility*. Pertinent to gastrointestinal disease, ghrelin has multiple gastrointestinal effects, including an increased rate of gastric emptying and a more rapid small intestinal transit [12, 13, 32], although there are no effects on colonic smooth muscle contraction [33–35]. Importantly, these GI effects occur even in the presence of vagotomy, suggesting a local action [36].

In addition to these effects of the acyl form of ghrelin, there appear to be effects of the des-acylated form. Though the des-acylated form of ghrelin does not bind to the GHSR-1a and does not have a known receptor, its administration has been noted to produce additional systemic effects as well, including adipogenesis [37].

## 3. IBD Pathophysiology and Treatment

The pathophysiology of both Crohn's disease and ulcerative colitis appears to begin at the local mucosal level with an exaggerated inflammatory response to intraluminal features such as commensal bacteria [1]. This local inflammation worsens to the point of producing erosions in the epithelium, leading to ulcerations and continued worsened inflammation. The local production of inflammatory mediators such as TNF-*α*, IL-6, and IL-1*β* results in a systemic inflammation [1]. In the case of ulcerative colitis, the ulcerated epithelium results in a high enough degree of inflammatory mediators and ulceration that it produces painful defecation and bloody stools and is usually noted before the process results in significant weight loss [38].

In the case of Crohn's disease, however, there can frequently be seen an insidious onset of disease that results in a catabolic state associated with decreased appetite [39] and a loss of fat mass [40]. During active disease, IBD can also result in endocrine abnormalities in humans and animals models of IBD that are likely related to both systemic inflammation and decreased levels of leptin [41–47].

Treatment of IBD has focused predominantly on decreasing inflammation. This is effectively accomplished by agents targeting local and/or systemic inflammation. This includes biologic medications such as anti-TNF-*α* antibodies (infliximab and adalimumab), immunomodulating agents such as azathioprine and methotrexate, and corticosteroids [2]. The goal in treatment is to induce a remission of local inflammatory activity. It is notable that a less frequently used approach utilizes increased enteral nutrition. Despite not as effective as a therapy as corticosteroids [48], this treatment approach is used as first-line therapy in some settings [3]. This approach results in a decrease in systemic inflammation [49] which is apparent by day 7 of treatment, which is notable given that changes in BMI may not be present until day 14 of treatment [50].

## 4. Ghrelin Levels in IBD

Given the conditions of decreased body weight and low levels of leptin, it is not surprising that ghrelin levels have been shown to be elevated in Crohn's disease Table 1 [51–54]. This includes elevations in levels of acyl ghrelin (elevated 28–329% above controls) [51, 52] as well as total ghrelin (elevated 60% above controls) [53]. Despite having a lower degree of weight loss, individuals with ulcerative colitis exhibit similar differences in acyl (28%) and desacyl (224%) ghrelin [51, 52]. Additionally, subjects whose IBD is in remission have lower levels than those with active disease [51, 53–55]. Although the stomach is the predominant source of ghrelin secretion in the body, an examination of ghrelin expression in colonic tissue revealed higher levels of ghrelin mRNA among patients with Crohn's disease and UC than among control patients, though the significance of this increase in expression is uncertain [6, 56].

While these elevations in ghrelin appear to be at least in part due to the presence of active disease, body weight—and particularly fat mass—further modulates ghrelin levels in the setting of experimental colitis. This was demonstrated following induction of colitis using trinitrobenzene sulfate (TNBS) in two groups of rats: a group with obesity induced by a high-fat diet and a rat strain that is resistant to the high-fat diet [57]. The diet-resistant rats weighed 30% less than the rats with diet-induced obesity and had leptin levels that were 75% lower. While levels of ghrelin were not significantly different between these groups in the absence of colitis, during TNBS colitis, the diet-resistant rats had ghrelin levels that were 30% higher than the rats with diet-induced obesity and colitis. Thus, elevated levels of ghrelin in colitis may be in part related to low levels of body fat or leptin, both of which have been previously shown to have inverse relationships with levels of ghrelin [16]. 

## 5. Use of Ghrelin in Disease Associated with Catabolism

Despite the elevated levels of ghrelin during active Crohn's disease, many of the processes affected by ghrelin (including increased appetite, increased adiposity, and decreased inflammation) exhibit the opposite of their expected response. This suggests that these elevations in ghrelin may be a physiologic response that continues to be countered by other processes. Ghrelin's effects on metabolism and appetite have led to investigations into its use as a treatment for diseases that involve excess catabolism and pathologic anorexia. The majority of these diseases have a pronounced component of a disease-associated cachexia [25]. 

Even though endogenous ghrelin levels are increased in diseases such as cancer [58], renal failure [59], and cardiac failure [60], administration of ghrelin or ghrelin analogues to animal models and humans with these conditions results in improvements in lean body mass and appetite and a decrease in circulating inflammatory cytokines [11, 28, 29, 61–65]. This is to say that although there is some element of ghrelin resistance in these settings, pharmacologic doses of GHSR-1a agonists (67–800 nmol/kg/d in animals; 2.4–3.6 nmol/kg/d in humans) have been able to overcome this resistance to some extent in these disease states [7]. While IBD is distinct from processes that result in cachexia, there also remain similarities between these states such as chronic inflammation, decreased appetite, and a loss of body weight. Given the efficacy of ghrelin in improving body weight and decreasing inflammation in other disease states, the potential use of ghrelin as a treatment in IBD was a logical extension of prior investigations.

## 6. Ghrelin as a Treatment for IBD

**Figure 1:**
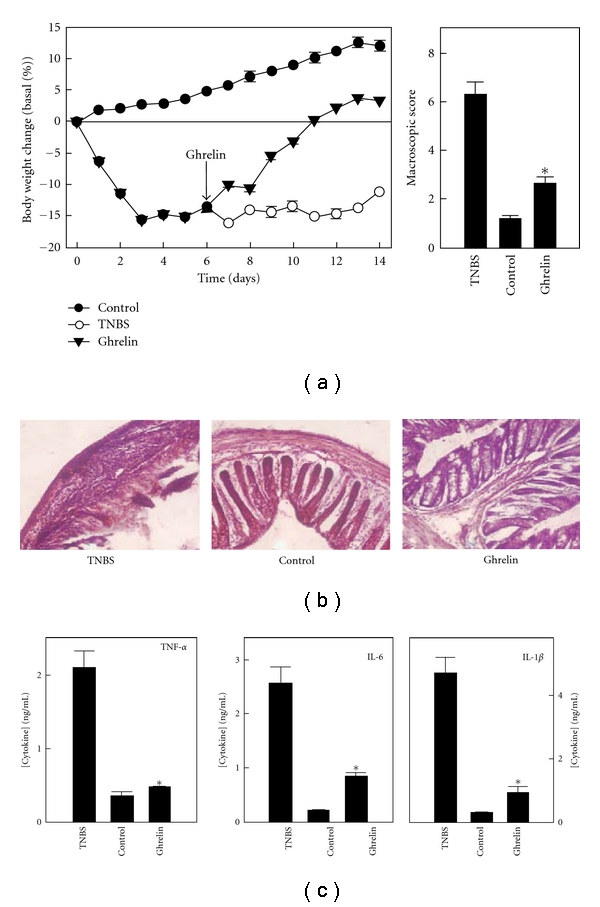
*Efficacy of ghrelin as a treatment for TNBS colitis.* For each set of experiments shown, TNBS colitis was induced via intrarectal injection on day 0 while control mice received 50% ethanol. Mice in (a) were given ghrelin as a rescue medication on days 6–9 of colitis and exhibited a rapid improvement in weight and in macroscopic score. Mice in (b) and (c) were given a single ghrelin injection 12 hours after TNBS treatment and had histology (b) and serum cytokines (c) examined on day 3 after treatment. *P < 0.05 versus TNBS (adapted from Gastroenterology 130: 1707–1720, used by permission).

Thus far experiments testing the effects of ghrelin treatment in the setting of IBD have only been performed on animal models of colitis. The most extensive investigations regarding the effects of ghrelin in IBD were performed by Gonzalez-Rey et al. In a set of experiments that primarily induced colitis via intrarectal administration of TNBS, these investigators demonstrated that ghrelin treatment produced a near-total amelioration of multiple findings of colitis, including weight loss, histological colitis score, survival, and myeloperoxidase activity in the colon (Figure 1) [5]. The effects of ghrelin treatment were most pronounced when given as a single 2 nmol dose (90 nmol/kg) via intraperitoneal (IP) injection 12 hours after TNBS administration. However, ghrelin was also effective when used as a rescue medication 6 days after induction of colitis, at which point repeated treatment with ghrelin (2 nmol/day, representing *∼*105 nmol/kg/day IP × 3 d) resulted in weight regain and a >50% reduction in macroscopic score (Figure 1(a)). Similarly, when using a dextran sodium sulfate (DSS) model of colitis, they found that when initiated on day 4 of DSS treatment ghrelin caused an improvement in body weight (compared to 20% weight loss in the untreated DSS group), a 67% decrease in disease activity, and a near normalization of colon myeloperoxidase activity.

Ghrelin treatment in this model also appeared to confer resistance to reactivation of disease. Mice treated with ghrelin 12 hours after initial TNBS administration were almost entirely resistant to a second TNBS administration 9 days later, as compared to mice treated with TNBS and saline, in whom a second dose of TNBS resulted in brisk deterioration [5]. This suggests long-term benefits following initial treatment.

Accompanying markers of disease activity, ghrelin treatment of TNBS colitis also resulted in suppression of local and systemic inflammation. By 3 days following induction of TNBS colitis, the single dose of 2 nmol of ghrelin given 12 hours after TNBS resulted in a decrease in multiple systemic inflammatory cytokines to near control levels, including TNF-*α*, IL-1*β*, and IL-6 (Figure 1(c)). In these experiments, levels of expression and quantity of inflammatory cytokines in the colons of ghrelin-treated animals were decreased almost to levels seen in noncolitic animals. These effects on cytokines may be due to direct immunological effects of ghrelin as opposed to merely being due to an absence of colitis. This is suggested by an increase in colonic quantities of IL-10, an anti-inflammatory cytokine, which in TNBS/ghrelin-treated mice was increased to levels 2-fold higher than seen in noncolitic mice and TNBS-saline-treated animals. Suppression of inflammatory cytokines was also demonstrated following ghrelin treatment in established disease. In these experiments, ghrelin was administered 6 days after induction of TNBS, resulting in a rapid return to near-baseline levels of TNF-*α*, IL-6, and INF-*γ* within 5 days of ghrelin treatment. Again noted in this setting was an increase in colonic levels of IL-10 levels.

Many of the findings of Gonzalez-Reys et al. regarding ghrelin administration in colitis were confirmed by Konturek et al. using a rat model of TNBS colitis [6]. In their experiments, daily administration of ghrelin at a dose of 20 ug/kg (5.9 nmol/kg/d) via IP injection starting 3 days after TNBS administration resulted in significant healing of colitis. Ghrelin treatment produced a 50% decrease in mean lesional area that was accompanied by a 20% increase in colonic blood flow. These researchers postulated that ghrelin's beneficial actions required activity of inducible nitric oxide synthase (iNOS), as pharmacologic inhibition of iNOS abrogated any effect of ghrelin administration in colitis. They also noted an increase in COX-2 protein in the colonic mucosa of rats treated with ghrelin, suggesting a potential additional mechanism for anti-inflammatory effects.

Alongside these studies demonstrating benefits to ghrelin treatment in animal models of colitis, another set of researchers—De Smet et al.—concluded the opposite regarding the role of ghrelin in experimental colitis [66]. These researchers used a DSS model of colitis in which noninbred Swiss mice were given 3% DSS in the drinking water for 5 days (followed by 5 days off DSS but with continued colitis). Mice were treated with IP injections of saline or ghrelin 100 nmol/kg twice daily, starting 8 hours after initial DSS exposure. These researchers found that ghrelin enhanced disease activity scores, increased colonic neutrophil infiltration, and increased colonic myeloperoxidase activity and IL-1*β* protein content. Interestingly, in addition to noting these worsened effects in mice treated with ghrelin, they also noted similar differences between wild-type mice and ghrelin knock-out mice with DSS colitis, with knock-out mice having improved tolerance of DSS colitis. They concluded that any ghrelin—including endogenous production—resulted in increased colonic inflammation in DSS colitis.

In many ways, it is difficult to interpret the reasons for contrasting findings between these sets of researchers. This is particularly true regarding the differences in ghrelin's effects in the setting of DSS colitis. In their ghrelin/DSS experiments, Gonzalez Rey and De Smet had differences in the dose of ghrelin (2 nmol (approximately 90 nmol/kg/d) on days 4 and 6 [5] versus 100 nmol/kg twice daily for 10 days [66]), differences in mouse strain (Balb/c [5] versus noninbred Swiss mice [66]), and differences in DSS regimen (5% DSS from Sigma [5] versus 3% DSS from MP Biomedicals [66])—each of which may have played a role. 

Perhaps the more significant contrast, however, is between the use of DSS colitis and TNBS colitis, which was used by Konturek et al. and was the predominant model used by Gonzalez-Rey et al. DSS colitis involves the administration in the drinking water of large, nonabsorbed polymers that irritate the colonic mucosa, causing epithelial ulceration and subsequent recruitment of inflammatory cells. This inflammatory response is not T-cell dependent [67]. Thus, while DSS colitis is a reasonable model for investigating aspects of local and systemic effects of colitis, it is not regarded as an optimal model for testing treatment of colitis. TNBS colitis requires helper T-cell response and in this sense bears similarities with human disease [68]. Konturek and Gonzalez-Rey used different species (rats versus mice), doses (5.9 nmol/kg/d versus 90 nmol/kg/d), and time courses (various treatment regimens), but reported similar improvements following ghrelin treatment of colitis. This gives early evidence of generalizability of ghrelin's effects in this model of colitis. Further investigation using other models—or trials in humans—has not been reported.

## 7. Ghrelin's Mechanism Related to IBD Effects

**Figure 2:**
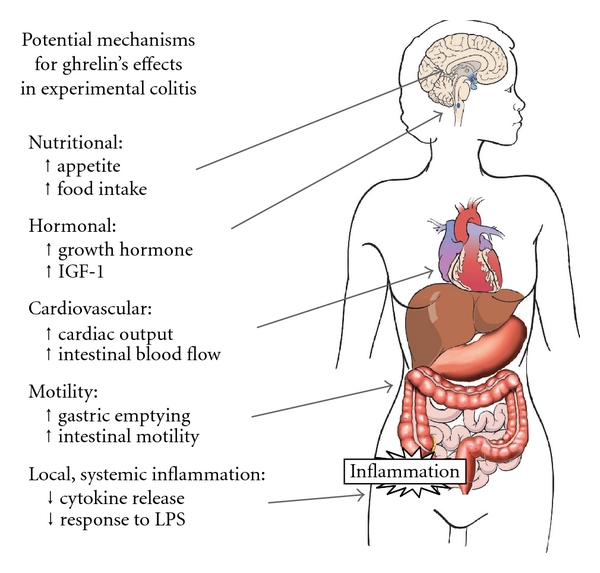
*Potential mechanisms of ghrelin action in colitis.* Ghrelin acts on the GHSR-1a in widespread tissues, causing several effects that might contribute to efficacy in features of IBD (adapted from Nature Clinical Practice 2: 459–466, used by permission).

The mechanism of ghrelin's effects in suppressing colitis in animal models is unclear, though multiple effects of ghrelin may play a role in the setting of IBD (Figure 2) [69].

### 7.1. Decreased Local and Systemic Inflammation

Ghrelin has been shown to decrease levels of inflammatory cytokines, when administered to isolated leukocytes and when given in models of disease. Systemic administration of anti-inflammatory agents such as infliximab in IBD has clearly decreased local disease activity as well as systemic activity. The decrease in systemic inflammation seen following ghrelin administration in these models of colitis could be a primary effect or due to a decrease in local disease activity mediated by another effect of ghrelin.

### 7.2. Increase in Growth Hormone Activity

None of the reports of ghrelin administration in colitis have included levels of growth hormone or IGF-1, though studies of GHS-1a agonist treatment in cachexia have reported increased levels of IGF-1 [70]. Use of growth hormone as a treatment for IBD has resulted in increased linear growth [46] but not an improvement in disease activity [71], suggesting that this is not a primary mechanism by which ghrelin improves colitis.

### 7.3. Increased Motility

Ghrelin's increase in intestinal motility and decrease in transit time could theoretically decrease exposure of intestinal mucosa to agents responsible for local inflammatory reaction. These agents could include products of commensal bacterial or—in the setting of animal models of colitis—treatment agents such as TNBS and DSS.

### 7.4. Increased Food Intake

As mentioned previously, enteral feeding continues to be used in some settings of human IBD. While increased food intake in the setting of ghrelin treatment is a plausible mechanism by which ghrelin may improve inflammatory bowel disease, none of the current reports on ghrelin administration have reported increases in food intake, even in cases of increased body weight.

### 7.5. Cardiovascular Effects

Konturek et al. noted an increase in colonic blood flow among colitic rats treated with ghrelin [6]. While ghrelin has had cardiovascular effects noted in multiple settings (including increased cardiac output and decreased blood pressure) [27], it is not certain whether these cardiovascular observations are the result of direct activity or indirect related to decreased disease. Konturek noted that the increase in blood flow was completely blocked during inhibition of iNOS, suggesting that either the direct or indirect action is mediated through production of nitric oxide.

## 8. Remaining Questions

Clearly many unanswered questions remain regarding the potential role of ghrelin as a treatment for IBD. Chief among these is whether ghrelin would have any benefit among humans—and if so, at what dose and route. Preliminary studies of ghrelin in humans with cachexia have been promising regarding the lack of significant side effects and the emergence of small molecule GHSR-1a agonists that have a longer half-life than ghrelin itself [25]. A key difference between IBD and these diseases, however, is that multiple effective treatments exist for the underlying pathophysiology of IBD, while cachexia remains without effective treatments. It is not at all clear that ghrelin administration would offer improvements beyond current treatments for IBD.

## 9. Conclusion

In conclusion, ghrelin is an endogenous hormone that is involved in appetite stimulation, growth hormone release, and gut motility and which appears to have anti-inflammatory properties. Though ghrelin levels appear to be elevated in Crohn's disease, treatment with exogenous ghrelin in animal models has been shown in some—but not all—studies to improve disease course. While this efficacy is promising, it remains to be seen whether ghrelin treatment in humans with IBD could overcome the adverse processes leading to appetite suppression, inflammation, and gut motility. Also uncertain are the mechanisms by which ghrelin may produce these effects. Clearly, more investigation is necessary.

## Figures and Tables

**Table 1:** Levels of acyl and total ghrelin among IBD subjects and healthy controls.

	Healthy controls	CD, active	CD, remission	UC, active	UC, remission	IBD, active	IBD, remission
Acyl ghrelin							

Ates et al. 2008 [51]	84 ± 14	110 ± 10***	75 ± 15	108 ± 11***	71 ± 13		
Karmiris et al. 2006 [52]	14.8 ± 3.0	49.4 ± 4.2^###^		48.2 ± 4.2^###^			

Total ghrelin							

Peracchi et al. 2006 [53]	203 ± 81.1					323.6 ± 119.2^∗∗∗, ###^	217.4 ± 64.9
Nishi et al. 2005 [54]		220.6 ± 98.8 NS	202.3 ± 86.4				
Alexandridis et al. 2009 [55]						402.4 ± 462.6	148.2 ± 59.6*

Statistical significance: active disease versus remission: *P < 0.05, ***P < 0.001; active disease versus controls: ^###^ *P* < 0.001; NS: not significant.
